# Comparison of tumor cell numbers and 22C3 PD-L1 expression between cryobiopsy and transbronchial biopsy with endobronchial ultrasonography-guide sheath for lung cancer

**DOI:** 10.1186/s12931-019-1162-3

**Published:** 2019-08-16

**Authors:** Ken Arimura, Mitsuko Kondo, Yoji Nagashima, Masato Kanzaki, Fumi Kobayashi, Kiyoshi Takeyama, Jun Tamaoki, Etsuko Tagaya

**Affiliations:** 1 Department of Respiratory Medicine, Tokyo Women’s Medical University, 8-1 Kawada-cho Shinjuku-ku, Tokyo, 162-8666 Japan; 2 Department of Surgical Pathology, Tokyo Women’s Medical University, Tokyo, Japan; 3 Department of Thoracic Surgery, Tokyo Women’s Medical University, Tokyo, Japan

**Keywords:** Cryobiopsy, The number of tumor cells, Programmed death ligand 1 expression, Endobronchial ultrasonography-guide sheath, Peripheral pulmonary lesions

## Abstract

**Background:**

We previously reported cryobiopsy (Cryo) with endobronchial ultrasonography-guide sheath (EBUS-GS) for peripheral pulmonary lesions (PPLs) provides significantly larger tissues than transbronchial biopsy (TBB) and provides high quantity and quality DNA for gene analysis by next generation sequencing. However, the tumor cell yields and programmed death ligand 1 (PD-L1) expression between each approach have not been compared. Here, we assessed the tumor cell numbers and PD-L1 expression for Cryo with EBUS-GS for PPLs and TBB in patients with lung cancer.

**Methods:**

Sixteen patients were enrolled in this prospective study from June to November 2017 at Tokyo Women’s Medical University Hospital. The number of tumor cells from a single biopsy, total number of tumor cells, average number of tumor cells, and 22C3 PD-L1 expression (≥ 50% and ≥ 1%) were compared between Cryo and TBB.

**Results:**

The numbers of tumor cells from a single biopsy, total numbers of tumor cells, and average numbers of tumor cells obtained by Cryo were significantly larger than those obtained by TBB (Cryo [means ± standard errors of the means]: 1321 ± 303.7, 1981 ± 411.7, and 1406 ± 310.3; TBB: 208.8 ± 38.24, 1044 ± 189.0, and 208.8 ± 37.81; *P* < 0.0001, *P* = 0.0474, *P* = 0.0006, respectively). PD-L1 ≥ 50% and ≥ 1% patients for Cryo were 18.8 and 56.3%, respectively, whereas those for TBB were 12.5 and 37.5%, respectively. The sensitivity, specificity, positive predictive value, negative predictive value, concordance, and κ coefficient based on Cryo for TBB were 66.7, 100, 100, 92.9, 93.8%, and 0.7647, respectively, for PD-L1 ≥ 50%; and 44.4, 71.4, 66.7, 50, 56.3%, and 0.1515, respectively, for PD-L1 ≥ 1%.

**Conclusion:**

Cryo with EBUS-GS may be a useful diagnostic approach for lung cancer, with advantages over TBB for gene analysis and whole exon sequencing. Particularly, it could contribute to patients taking pembrolizumab as first-line therapy when PD-L1 was negative by evaluating TBB specimens. It could also provide ample tissue for PD-L1 expression analysis in addition to accurate diagnosis and gene analysis.

## Background

Lung cancer is the most prevalent cause of cancer-related death worldwide. Peripheral pulmonary lesions (PPLs) suspicious for lung cancer have been detected at high frequency following the increased utilization of computed tomography (CT) of the chest. The 3rd edition of the American College of Chest Physicians guidelines recommends using endobronchial ultrasonography (EBUS) for PPLs [1]. Transbronchial biopsy (TBB), transbronchial needle aspiration, and brushing with EBUS-guide sheath (EBUS-GS) have been recognized as useful strategies for the diagnosis of PPLs [2–9]. In addition to diagnostic applications, it is also recommended to validate the programmed death ligand 1 (PD-L1) expression [10, 11] and maximize the volume of tissue for phenotyping and genotyping [12]. However, tissues from PPLs obtained by conventional biopsy are generally small [13]. Therefore, cryobiopsy (Cryo) with EBUS-GS may be a useful tool for overcoming this problem. Cryo with EBUS-GS has been shown to be a safe and useful tool for PPLs suspicious of lung cancer [14, 15]. We previously reported that Cryo with EBUS-GS yields significantly larger tissues than TBB and provides high quantity and quality DNA for gene analysis by next generation sequencing. Furthermore, Cryo with EBUS-GS provides a high concordance between rapid on-site evaluation and the final diagnosis [14]. However, comparisons of tumor cell numbers and PD-L1 expression in tissues obtained between Cryo and TBB have been unknown. Because Cryo with EBUS-GS yields larger tissues than TBB, we hypothesized that Cryo with EBUS-GS may be able to have more tumor cells and may therefore be more suitable for evaluation of PD-L1 expression compared with TBB. Therefore, the purpose of this study was to assess the tumor cell numbers and PD-L1 expression obtained by Cryo and TBB.

## Methods

### Ethical considerations

This was a prospective study approved by the Institutional Review Board of Tokyo Women’s Medical University Hospital (date of approval: April 19, 2017; approval number: 170404). Informed consent was obtained from all patients before enrollment in this study.

### Patient population and study design

The eligibility criteria and exclusion criteria were as previously described [14]. Eligible patients were over 20 years old and had PPLs suspicious of lung cancer. Patients were excluded from the study if they showed any of the following features: bleeding predisposition, platelet count < 20,000/mm^3^, pregnancy, active infection, respiratory insufficiency, lesions less than 2 cm from the pleura, obvious blood vessels adjacent to EBUS over 0.5 cm, and refusal to participate in the study [14]. In total, 23 patients underwent Cryo with EBUS-GS at Tokyo Women’s Medical University Hospital, and 16 patients who were given a diagnosis by biopsy were enrolled in this study.

PPLs were defined as abnormal and solid shadows in the pulmonary parenchyma, which were not identified with bronchoscopy [14, 16], and ground glass nodules were excluded. All PPLs were identified with CT or 18F-fluorodeoxy glucose-positron emission tomography prior to Cryo with EBUS-GS [14]. The lesion size was measured at the largest diameter [14]. Each patient was underwent brushing, TBB, and Cryo in this series.

### Procedures

The procedures used in this study were described previously [14]. A flexible fiber bronchoscope (BF-1TQ290; Olympus, Tokyo, Japan), 20-MHz radial EBUS probe (UM-S20-20R; Olympus), guide sheath (SG-201C; Olympus), brush (BC-202D-2010; Olympus), forceps (FB-231D; Olympus), and 1.9 mm cryo probe (CRYO2; ERBE, Tuebingen, Germany) were employed [14]. Thrombin (Liquid Thrombin MOCHIDA Softbottle 10,000; Mochida Pharmaceutical, Tokyo, Japan) and balloon catheter (B5-2C; Olympus) were prepared in case of mild or severe bleeding [14]. Local anesthesia with 1% lidocaine for nebulizing, 2% lidocaine bolus to the bronchus, intravenous injection of 2–2.5 mg of midazolam, and intra-muscular injection of 35 mg pethidine hydrochloride for conscious sedation were used during the procedures [9, 14]. The blood pressure, oxygen saturation, pulse rate, and electrocardiography of all patients were monitored in this study [9, 14].

### Sampling methods

Sampling methods were previously described [14]. Briefly, all patients were intubated with an endotracheal tube (7.5 mm TaperGuard; COVIDEN, MN, USA) to simplify the insertion and removal of the bronchoscope [14]. After confirmation of PPLs by EBUS and fluoroscopy, the internal EBUS was removed, leaving behind GS [3, 4, 14]. EBUS was withdrawn when PPLs were not identified, and a curette was inserted into GS to move into the appropriate bronchial segment [3, 14]. The curette was withdrawn, and EBUS was reinserted into GS to confirm PPLs under fluoroscopy [3, 14]. Brushing, TBB, and Cryo were performed in this series to obtain cytological materials and tissues [14]. Brushes for brushing were inserted into GS two times consecutively to obtain cytological materials. The forceps for TBB were inserted into GS 5 times to obtain tissues [14]. After the Cryo probe was wetted with alcohol cotton (STERI COTTOα; Kawamoto, Osaka, Japan) to facilitate smooth insertion into GS, the Cryo probe was inserted into GS and frozen with carbon dioxide for 3–5 s to about − 70 °C 1 or 2 times [14]. Subsequently, the Cryo probe was withdrawn together with GS and bronchoscope and then thawed in saline to obtain histological tissue [14]. The brush, forceps, and Cryo probe were washed with saline for cytological evaluation, cell blocks, bacterial cultures, acid-fast bacteria cultures, and polymerase chain reaction [14]. Each patient underwent chest radiography to assess potential complications 1 h after bronchoscopy [9, 14].

### Sampling process and diagnosis

Sampling process and diagnosis were performed as previously described [14]. The tissues obtained by Cryo were cut in half [14]. One of the tissues obtained by Cryo and the tissue by TBB were immediately fixed with 20% formalin, stained with hematoxylin and eosin (HE) staining and immunohistochemistry (IHC) staining for histological evaluation and PD-L1 expression [14]. The other tissue obtained by Cryo was immediately frozen at − 80 °C for DNA sequencing analysis [14]. Every pathological specimen was evaluated by an experienced pathologist to reach a diagnosis [14].

### Evaluation of tumor cell numbers and PD-L1 expression

After the pathologist reached a diagnosis, the number of tumor cells was counted manually by one cytoscreener and one pulmonologist in a blinded manner using HE staining slides. Then, the average number of tumor cells was calculated.

After sectioning of samples to 4–5 μm, PD-L1 staining was performed with 22C3 antibodies (rabbit monoclonal, clone 22C3; Agilent Dako, Glostrup, Denmark) using autostainer (Autostainer Link 48, Agilent Dako). PD-L1 positivity was defined as membranous staining in at least 1% of cells [10], regardless of staining intensity and proportion in the membrane. PD-L1 was evaluated by experienced pathologist, and the cut off values were classified as ≥50% and ≥ 1%. The number of tumor cells by a single biopsy, total number of tumor cells, average number of tumor cells, and PD-L1 expression for each patient were compared between Cryo and TBB.

### Data analysis

Data analysis was carried out using Graph Pad PRISM (GraphPad Software, La Jolla, CA, USA). T-tests were used to compare the numbers of tumor cells between Cryo and TBB. Differences with *P* values of less than 0.05 were considered statistically significant. The odds ratio (OR), sensitivity, specificity, positive predict value (PPV), negative predict value (NPV), concordance, and Cohen’s kappa (κ) coefficient based on Cryo for TBB were used to assess PD-L1 expression. The concordance rate was classified according to κ value as slight agreement (0–0.20), fair agreement (0.21–0.40), moderate agreement (0.41–0.60), substantial agreement (0.61–0.80), or almost perfect agreement (0.81–1.0) [17].

## Results

### Baseline characteristics

Patient characteristics, including the number, gender, median age, smoking history, median size of PPLs, tumor, nodes, metastasis (TNM) stage, and final diagnosis by bronchoscope, are summarized in Table 1. We diagnosed 10 adenocarcinomas, 4 squamous cell carcinomas, 1 small cell lung cancer, and 1 metastatic lung tumor.

**Table 1:** Patient characteristics

Patient characteristics	Value
Patients no.	16
Male/Female no.	14/2
Median age (range)	69 (46–82)
Smoking history no. (yes/no)	14/2
Median size (cm) of PPLs (range)	3.9 (1–8.1)
TNM stage no. (%)	
I	3 (18.8)
II	3 (18.8)
III	5 (31.3)
IV	5 (31.3)
Histological subtype no. (%)	
Adenocarcinoma	10 (62.5)
Squamous cell carcinoma	4 (25)
Small cell lung cancer	1 (6.25)
Metastatic lung tumor	1 (6.25)

*PPLs* peripheral pulmonary lesions, *TNM* tumor, nodes, metastasis

### Comparison of tumor cell numbers between Cryo and TBB specimens

**Fig. 1:**
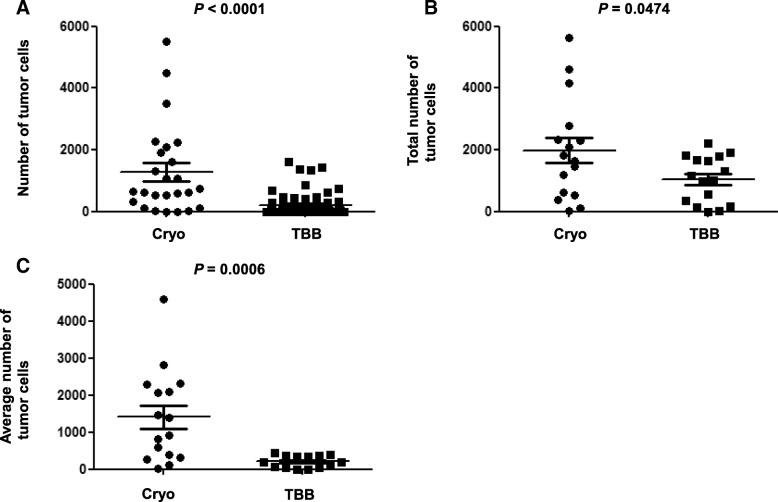
Comparison of the numbers of tumor cells between Cryo and TBB. **a**, The number of tumor cells obtained by a single biopsy. 1321 ± 303.7 (mean ± SEM) for Cryo, 208.8 ± 38.24 for TBB (95% CI: 756.8–1467, *P* < 0.0001). **b**, Total number of tumor cells obtained by each biopsy. 1981 ± 411.7 for Cryo, 1044 ± 189.0 for TBB (95% CI: 11.79–1862, *P* = 0.0474). **c**, Average number of tumor cells obtained by each biopsy. 1406 ± 310.3 for Cryo, 208.8 ± 37.81 for TBB (95% CI: 558.6–1835, *P* = 0.0006). Cryo, cryobiopsy; TBB transbronchial biopsy; SEM, standard error of the mean; CI, confidence interval

Comparisons of tumor cell numbers between Cryo and TBB are shown in Table 2. The number of tumor cells obtained from a single biopsy by Cryo was significantly larger than that by TBB (Cryo [mean ± standard error of the mean]: 1321 ± 303.7; TBB: 208.8 ± 38.24; 95% confidence interval [CI]: 756.8–1467, *P* < 0.0001, Fig. 1a). The total number of tumor cells obtained by Cryo was significantly larger than that obtained by TBB (Cryo: 1981 ± 411.7; TBB: 1044 ± 189.0; 95% CI: 11.79–1862, *P* = 0.0474, Fig. 1b). Furthermore, the average number of tumor cells obtained by Cryo was also significantly larger than that obtained by TBB (Cryo: 1406 ± 310.3; TBB: 208.8 ± 37.81; 95% CI: 558.6–1835, *P* = 0.0006, Fig. 1c).

**Table 2:** Comparison of the number of tumor cells from a single biopsy, total number of tumor cells, average number of tumor cells, and PD-L1 expression between Cryo and TBB

Cryo						TBB							
Case	No. of tumor cells		Total no.	Average no.	PD-L1 (%)	No. of tumor cells					Total no.	Average no.	PD-L1 (%)
1	4601		4601	4601	10	634	402	337	332	198	1903	380.6	5
2	381		381	381	0	1622	29	0	0	0	1651	330.2	0
3	25		25	25	0	871	302	140	0	0	1313	262.6	0
4	2080		2080	2080	100	143	98	76	10	8	335	67	100
5	115		115	115	10	3	0	0	0	0	3	0.6	0
6	3509	642	4151	2075.5	10	63	50	25	22	0	160	32	0
7	1467		1467	1467	40	14	0	0	0	0	14	2.8	0
8	532	0	532	266	0	1340	449	30	12	0	1831	366.2	0
9	2248	541	2789	1394.5	0	124	25	0	0	0	149	29.8	0
10	2321		2321	2321	10	334	294	157	124	96	1005	201	0
11	618	0	618	309	5	471	217	169	119	0	976	195.2	0
12	1621	31	1652	826	0	331	222	0	0	0	553	110.6	0
13	5520	121	5641	2820.5	90	1380	137	90	61	7	1675	335	100
14	2301		2301	2301	70	1431	471	145	90	66	2203	440.6	10
15	625	578	1203	601.5	0	311	248	202	198	187	1146	229.2	10
16	1075	740	1815	907.5	0	750	675	302	58	0	1785	357	10

*Cryo* cryobiopsy, *TBB* transbronchial biopsy, *PD-L1* programmed death ligand 1

### Comparison of PD-L1 expression between Cryo and TBB specimens

**Fig. 2:**
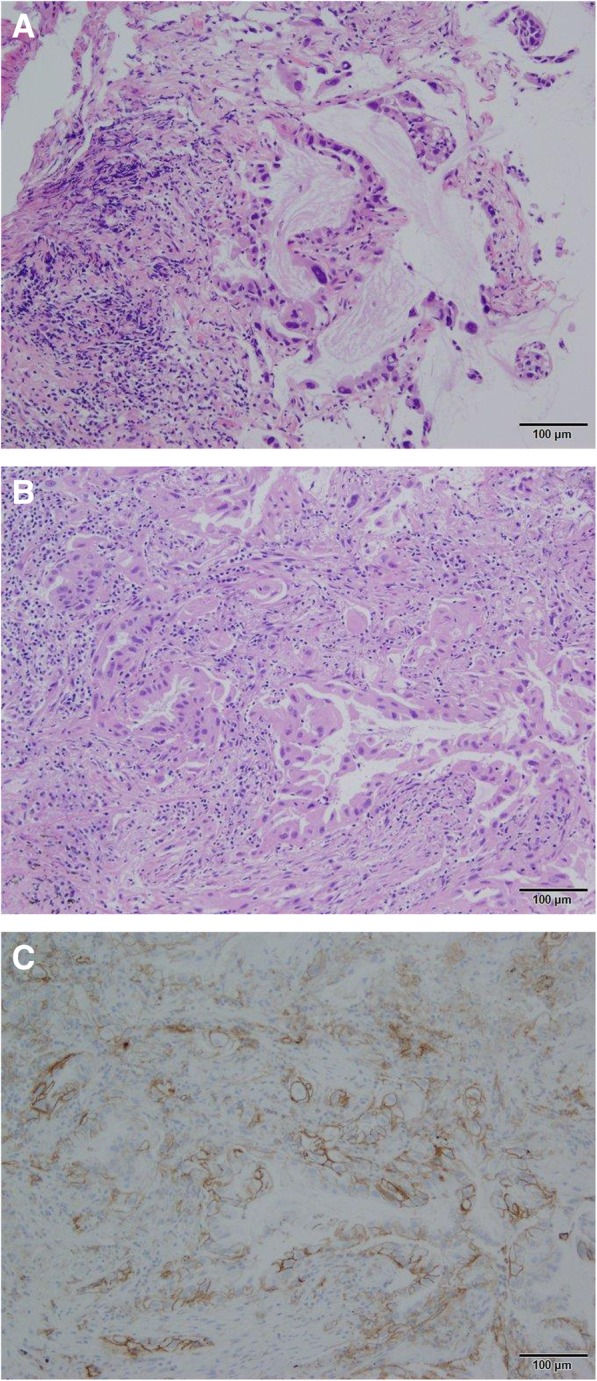
Representative image of HE staining for TBB and Cryo and PD-L1 ≥ 50% with the same patient (Adenocarcinoma 10×). **a**, HE staining for TBB specimens. **b**, HE staining for Cryo specimens. **c**, PD-L1 ≥ 50% for Cryo specimens. HE, hematoxylin and eosin; PD-L1, programmed death ligand 1; TBB, transbronchial biopsy; Cryo, Cryobiopsy

Comparisons of PD-L1 expression between Cryo and TBB are shown in Table 2. Representative image of HE staining for TBB and Cryo and PD-L1 ≥ 50% for Cryo with the same patient are shown in Fig. 2. PD-L1 ≥ 50% was observed in 18.8% of patients for Cryo and 12.5% of patients for TBB. PD-L1 ≥ 1% was observed in 56.3% of patients for Cryo and 37.5% of patients for TBB. The OR, sensitivity, specificity, PPV, NPV, concordance, and κ coefficient were 45 (95% CI: 1.394–1452), 66.7% (0.094–0.992), 100% (0.753–1), 100% (0.158–1), 92.9% (0.661–0.998), 93.8% (0.698–0.998), and 0.7647 (0.288–1), respectively, for PD-L1 ≥ 50% and 2 (0.244–16.37), 44.4% (0.137–0.788), 71.4% (0.290–0.963), 66.7% (0.223–0.957), 50% (0.187–0.813), 56.3% (0.299–0.803), and 0.1515 (0–0.608), respectively, for PD-L1 ≥ 1% (Table 3).

**Table 3:** Comparison of OR, sensitivity, specificity, PPV, NPV, concordance, and κ coefficient with 95% CI between Cryo and TBB according to PD-L1 expression

	PD-L1 ≥ 50% (95% CI)	PD-L1 ≥ 1% (95% CI)
OR	45 (1.394–1452)	2 (0.244–16.37)
Sensitivity	66.7% (0.094–0.992)	44.4% (0.137–0.788)
Specificity	100% (0.753–1)	71.4% (0.290–0.963)
PPV	100% (0.158–1)	66.7% (0.223–0.957)
NPV	92.9% (0.661–0.998)	50% (0.187–0.813)
Concordance	93.8% (0.698–0.998)	56.3% (0.299–0.803)
κ coefficient	0.7647 (0.288–1)	0.1515 (0–0.608)

*OR* odds ratio, *PPV* positive predict value, *NPV* negative predict value, *CI* confidence interval, *Cryo* cryobiopsy, *TBB* transbronchial biopsy, *PD-L1* programmed death ligand 1

### Adverse events

There were no clinically serious adverse events, except mild bleeding in 4 cases; all cases required endoscopic procedures with thrombin [14].

## Discussion

In this study, we described the excellent results of Cryo with EBUS-GS for PPLs. To the best of our knowledge, no other studies have reported comparisons of tumor cell numbers and PD-L1 expression between Cryo and TBB with EBUS-GS for PPLs. This report provides evidence about comparison of tumor cell numbers and 22C3 PD-L1 expression using Cryo with EBUS-GS.

In our study, the number of tumor cells from a single biopsy, total number of tumor cells, and average number of tumor cells obtained by Cryo were significantly larger than those obtained by TBB. Cryo with EBUS-GS had the advantage of yielding significantly larger specimens than TBB, as we previously reported [14, 15]. The volume obtained by Cryo was about 26 times greater than that obtained by TBB [14]. Therefore, the higher volume was expected to contribute to the significant differences in the number of tumor cells. Not only did Cryo yield more tumor cells than TBB but Cryo also showed higher total and average numbers of tumor cells, suggesting that it may be appropriate to perform Cryo 1 or 2 times for PPLs suspicious of lung cancer. In addition, performing Cryo 1 or 2 times would yield more DNA for subsequent analyses of lung cancers because the numbers of tumor cells were significantly larger than that obtained by TBB, despite performing TBB 5 times. Cryo specimens may be more appropriate for analyzing gene mutations and performing whole exon sequencing compared with TBB specimens. Furthermore, despite being cut in half and the one was used for gene mutation analysis [14], even the other half of the specimen was sufficient for evaluation of HE staining and PD-L1 expression. Notably, for PD-L1 ≥ 50%, we found high specificity (100%), PPV (100%), NPV (100%), and concordance (93.8%) and substantial agreement (0.7647) for κ coefficient. In contrast, for PD-L1 ≥ 1%, we found low sensitivity (44.4%), NPV (50%), and concordance (56.3%) and slight agreement (0.1515) for κ coefficient.

Some studies have assessed the concordance rate of PD-L1 expression between resected tissues and biopsy samples [18–20] or tissue microarrays [21]. One study using a specific hybrid IHC score with 4059 antibody showed good concordance between resected samples and TBB for PD-L1 expression [18]. Another study using positive/negative IHC scores with EPR1161 (2) antibody showed moderate concordance [19]. Similarly, we demonstrated high specificity, PPV, NPV, and concordance and substantial agreement for κ coefficient between Cryo, which showed a significantly larger volume such as resected specimen than TBB, and TBB for PD-L1 ≥ 50%. In contrast, we observed low sensitivity, NPV, and concordance and slight agreement for κ coefficient for PD-L1 ≥ 1%. We hypothesized that the reasons for this discrepancy between previous reports and our results with regard to PD-L1 ≥ 1% may be related to the use of different antibody, various scoring systems, and heterogeneity of PD-L1 expression.

Some immune checkpoint inhibitors have been proved to be effective for lung cancer treatment as first-line monotherapy [11, 22], first-line combination therapy [23–25], or second-line therapy [10, 26, 27]. However, pembrolizumab is the only immune checkpoint inhibitor found to be effective as a first-line monotherapy according to the proportion of PD-L1 expression. 22C3 antibody, which are regarded as a companion diagnostics, are associated with pembrolizumab. Accordingly, in this study, we used 22C3 antibody to detect PD-L1 expression. Importantly, some studies describing PD-L1 expression have found it different with various antibodies [28, 29], and various antibodies have been shown to have different cut-off values for PD-L1 expression [22–27]. Furthermore, some studies described the intra- and inter-tumor heterogeneity of PD-L1 expression [30–32]. Indeed, heterogeneity is the one of the reasons we had 2 false-positive cases for PD-L1 ≥ 1%. Moreover, Cryo yielded larger specimens [14] and higher tumor cell numbers than TBB. These reasons support our above interpretations and may explain the differences in results for PD-L1 ≥ 1% between previous studies [18, 19] and our current findings.

Our results regarding PD-L1 expression could contribute to patients taking pembrolizumab as first-line therapy [22] when PD-L1 was negative by evaluating TBB specimens. It could be reliable for evaluating PD-L1 expression to use Cryo specimens to prevent from leading to misclassification. Moreover, we showed that Cryo specimens had the advantages of not only providing tissues for accurate diagnosis and DNA for gene analysis for personalized therapeutic strategy [14] but providing ample tissue for evaluating PD-L1 expression.

This study had several limitations. First, it was performed at a single institution with a small number of patients and did not apply a randomized control design to validate the results. Second, we compared tumor cell numbers and PD-L1 expression between Cryo and TBB. Thus, a comparison of PD-L1 expression between Cryo and resected tissues should be performed in future studies. Third, although we used a smaller Cryo probe (1.9 mm), performing Cryo with a larger probe (2.4 mm, CRYO2; ERBE) may have yielded even larger tissues and more tumor cells for evaluating gene analysis and PD-L1 expression. However, such an approach may also cause clinically significant complications. The optimal size of Cryo probe still remains unknown.

## Conclusion

Cryo with EBUS-GS for PPLs is a useful diagnostic strategy. The number, total number, and average number of tumor cells obtained by Cryo were significantly larger than those obtained by TBB. Thus, this approach may be more appropriate for analyzing gene mutations and whole exon sequencing compared with TBB. These results could contribute to patients taking pembrolizumab as first-line therapy when PD-L1 was negative by evaluating TBB specimens. Cryo specimens could have an advantage of providing ample tissue for evaluating PD-L1 expression in addition to providing tissue for accurate diagnosis and DNA for gene analysis. Further studies with larger cohorts are needed to validate these results.

## Data Availability

The dataset supporting the conclusions of this study is presented in this manuscript. The clinical detail dataset is available from the author and corresponding author, but has not been made publicly available.

## Abbreviations

Cryo with EBUS-GS: Cryobiopsy with endobronchial ultrasonography using a guide sheath
PD-L1: Programmed death ligand 1
PPLs: Peripheral pulmonary lesions
TBB: Transbronchial biopsy
